# The Disguises of Fever

**Published:** 1897-07

**Authors:** 


### THE DISGUISES OF FEVER.

The older we grow the less we know. That at any rate is what I feel at times when I come up against the blank wall of a case of fever; is it typhoid, malaria or remittent? Now it is often a very difficult matter to make a satisfactory diagnosis early in typhoid fever, and the diagnosis is sometimes not made. We have had, I think, out of about 530 cases, with I forget how many deaths, two cases in which the diagnosis was made by Dr. Welch in the pathological laboratory and not made in the wards. There are a great many cases during the year in the country in which the diagnosis is never made correctly *intra vitam*, but only post-mortem. The doctor is lead astray in the diagnosis of typhoid in three main directions; first, its localization may deceive him. Typhoid fever may have a text-book symptomatology, or it may have no single suggestion of anything given in the text-book. It may, in the first place, begin with such marked cerebro-spinal manifestations that the physician is thrown off at once. It may not enter into his mind during a ten or twelve days' attendance upon a case that he is dealing with anything other than cerebro-spinal meningitis, and a certain number of the cases of cerebro-spinal meningitis reported by Dr. McShane, I will not say how many, but I would like to say a great many, are typhoid fever, with cerebro-spinal localization.

Beginning with headache, marked retraction of the head and neck, and in fact not a single feature of cerebro-spinal meningitis lacking, it is so clear to you that you do not care to look for the spots or examine the spleen. I am not thinking of theoretical cases, but cases which I have followed myself not only to the clinic, but to the autopsy room. I have records of some three or four cases in which a diagnosis of cerebro-spinal meningitis is made, but the autopsy clinched the diagnosis of typhoid. We have had one case here in which not only were the symptoms suggestive, but in addition to the spasm of the muscles of the neck and back he had clonic contractions of the head and arm.

Another localization is in the lungs or pleura. You see the case as one of pneumonia. The patient's first indication is a stitch in the side; you have a friction murmur and you are thrown off your guard entirely. The pleurisy cases are not so common as the pneumonia. We have had one well-marked case in which the symptoms were characteristic of pleurisy, and it was admitted and treated so for one week. The pneumonia cases begin with a pain in the side, rusty expectoration, high temperature, tubular breathing and indications of the envolvement of a lobe or the entire lung, and are more deceptive. You give a diagnosis of pneumonia; so it is, but about the tenth day, when you expect the crisis, it does not come; the temperature develops, the abdomen becomes tender and you have the extreme pleasure of telling the family that typhoid symptoms are developing. Now it is a blessing that Louis invented the name "typhoid symptoms." One of our errors of diagnosis was a case of that kind—an old man admitted with all the symptoms of pneumonia. It did not enter our minds for a moment that he had anything else than pneumonia until Dr. Welch examined him and then it was quite evident that he had typhoid fever.

Another mode of disguise is acute nephritis. That is fortunately rare, for the cases are almost invariably diagnosed as acute nephritis and not considered typhoid until a week or ten days have elapsed. Those are the chief errors of diagnosis and it is a point that I often find practitioners have not had their attention sufficiently called to ; namely, that typhoid may begin this way.

There are certain conditions that simulate typhoid fever. The simple continued fever, about which we have heard so much, is a fever that runs a week or ten days. You are safe to follow Murchison's opinion that the fever with local manifestations persisting ten days in a temperate latitude is likely to be typhoid. You must bear in mind that there are cases of typhoid with fever as the single manifestation, no swelling of the abdomen, no headache, patient feeling well and anxious to get up and you may have the diagnosis demonstrated in a very unpleasant way ; the patient being allowed to get up perforation may occur and hemorrhage follow, as I saw in a case recently. We must have had six or seven cases in the past seven years in which the diagnosis of the fever was not made in the preliminary fever for which the patient came into the hospital. The temperature dropped two or three days after coming into the hospital, a period of apyrexia of three or four days and then the original disease was determined by the onset of a relapse.

There is only one disease in this latitude with which typhoid is likely to be confounded and that is the estivo-autumnal intermittent fever. Now when I tell you that only a few months ago we treated a case for five or six days, the blood examination had been made, not by Dr. Thayer or myself, I might remark, but it was made and it was not until the sixth day that we could determine that we had been tubbing and treating a case of malaria ; you will understand that the similarity is very striking. In the first place take the mode of onset ; as a rule in typhoid fever there is a much longer period of malaise. The patient is out of sorts for a long period and epistaxis occurs frequently. Herpes is a complication of typhoid and it is one of the commonest in malaria.

Chills and chilly sensation are very common in both typhoid and malaria, the number of cases of typhoid beginning with chills being very considerable. Positive chills occurring at intervals of two or three days are more common, of course, in malaria, but if you take in consideration the chilly feelings they occur in about 50 per cent. of the cases of typhoid. There is a very great difference if you see the cases early, as you do generally in private practice, in the first or second week of typhoid or malaria. In malaria at the onset there are positive intermissions in the first week ; in typhoid there are none; it is a gradual slope upwards. In the second week the difference is very marked. There is no single disease in which so steady a fever is present as in typhoid. We have now dozens of charts, even from cases under treatment by cold baths, in which the diurnal range did not exceed one and a half or two degrees.

Examination of the blood is, of course, an important point, particularly now since the Vidal serum diagnosis method. In a certain number of cases of malaria, remittent and continued fevers, the examination of the blood has to be made repeatedly and often at short intervals, for the parasites are not present in the circulating blood in large numbers. It is very important that those who are to make examinations of the blood in malaria should have proper preliminary training.

Is there a typho-malarial fever? Yes, in the brains of the doctors, but not in the bodies of the patients. There is no combined hybrid disease and it is only due to Woodward to say that he did not recognize a hybrid disease. Typho-malaria is a villainous name and should be banished from our vocabularies and no doctor should ever use it, particularly to his patients. It gives a man a wrong sense of security and the doctor wastes a lot of good medicine, a lot of quinine, for instance, because he thinks there is some symptom that points to malaria. I am happy to say that cases of typho-malaria are disappearing slowly from the health reports ; they ought to be banished entirely. Chills, as I told you, occur frequently in the outset of a disease and they may occur throughout the course of a typhoid fever. The State boards of health hereafter should return to every physician who sends in a diagnosis of typho-malaria his blank and ask for something better. It is too late in the day, gentlemen, to make that diagnosis.—Dr. Wm. Osler in the *Maryland Medical Journal.*

